# Food web structure and ecosystem multifunctionality in a subsidized coastal ecosystem

**DOI:** 10.1038/s41598-025-25395-5

**Published:** 2025-11-07

**Authors:** Kyle A. Emery, Jenifer E. Dugan, David M. Hubbard, J. Carter Ohlmann, Jessica R. Madden, Robert J. Miller

**Affiliations:** 1 Marine Science Institute, University of California, Santa Barbara, Santa Barbara, CA 93106 USA; 2 Department of Geography, University of California, Los Angeles, Los Angeles, CA 90095 USA; 3 Earth Research Institute, University of California, Santa Barbara, Santa Barbara, CA 93106 USA

**Keywords:** Detritus, Sandy beach, Kelp forest, Foundation species, Wrack, Biodiversity, Ocean sciences, Food webs, Ecosystem ecology

## Abstract

The structure and functioning of all ecosystems face growing threats, ranging from local development to global climate change. Ecosystems with little to no in situ primary production may be disproportionately dependent on resources from other ecosystems. We examined the role of basal resource availability on community structure and ecosystem multifunctionality of sandy beaches. We hypothesized that substantial marine macrophyte wrack inputs from productive nearshore ecosystems, like kelp forests, would drive recipient beach ecosystem structure and function. Using piecewise structural equation modeling, we found wrack abundance had a strong positive effect on beach food web diversity and biomass of detritivorous and predatory macroinvertebrates as well as ecosystem multifunctionality, an integrative measure of ecosystem functioning. Resource inputs and biodiversity both explained ecosystem multifunctionality, but the role of biodiversity was strongly underpinned by wrack inputs. The diversity and abundance of top predators, shorebirds, responded similarly to resource availability. Our findings suggest the influence of marine foundation species, such as giant kelp, can extend to recipient ecosystems as detrital subsidies. The highly coupled nature of these coastal ecosystems increases the likelihood that negative impacts to donor ecosystems will cascade to affect the structure and function of subsidized recipient ecosystems.

**Supplementary Information:**

The online version contains supplementary material available at 10.1038/s41598-025-25395-5.

## Introduction

Biodiversity, community structure, food webs, and ecosystem functions are increasingly threatened across scales ranging from local impacts of human development to the global consequences of a changing climate^1,2^. Biodiversity and other drivers, including resource availability, may determine how the structure and function of ecosystems respond to this change^3,4^. Community structure and function are intrinsically linked, a multitude of studies have demonstrated this relationship using the impacts of climate warming, extinction, and more in both natural and experimental systems^5–8^. Climate and anthropogenic-driven changes can profoundly impact ecosystem functioning and community composition across multiple scales of observation^9^, highlighting the need to quantify the drivers of variation in these ecosystem attributes. Constraining the role of variability in ecosystem dynamics will enhance our ability to project changes in ecosystem structure and function due to natural and anthropogenic disturbances^10^.

Ecosystem function is inherently multifaceted, encompassing the processes and properties of biotic systems and their maintenance^11^. Critical ecosystem functions span the processes of primary and secondary production, community respiration, decomposition, nutrient cycling, nutrient retention, and system-level properties such as resistance to invasion^11^. Biodiversity, which can enhance primary production and other functions by maximizing niche space occupation via complementarity and facilitation among species, can be a key factor influencing ecosystem functioning^12^. The effect of biodiversity on ecosystem functioning has been most often estimated from experimental studies using microorganisms^13,14^ and manipulated plant communities^15,16^, but is increasingly being measured in natural settings^17,18^. In natural systems, however, the strength and direction of responses of ecosystem functions to drivers, including biodiversity, can vary significantly^19–21^. Competitive interactions, redundancy, or the abundance of high-functioning species, rather than diversity per se, can underlie this variability^22–24^.

While experimental results suggest that increasing biodiversity generally enhances ecosystem functioning as well as the overall stability of ecosystem functions^25^, most studies use individual functions as proxies for describing or quantifying overall ecosystem functioning. This approach can be misleading because of potential trade-offs among different functions and the higher level of redundancy within versus across functions^26^. An ecosystem multifunctionality approach that quantitatively combines several ecosystem functions can provide a more integrated index of functioning and help account for uncertainty in the estimates of single functions^27,28^. However, the lack of robust evaluations of ecosystem multifunctionality in more complex, multitrophic systems^21,29^ and in natural communities^17^ has hindered a more general understanding of the strength and importance of biotic and abiotic drivers of ecosystem functions in nature.

In natural systems, the availability of basal resources including habitat, net primary production, or subsidies can be the primary driver of ecosystem functioning rather than biodiversity^30–32^. In fact, ecosystem productivity has long been recognized as a driver of biodiversity^33^, but recent evidence has pointed to a more complex relationship between resources, productivity (i.e., biomass or abundance of organisms), and biodiversity^34–36^. Subsidized ecosystems are characterized by inputs of allochthonous organic matter or nutrients which based on the magnitude of subsidy received, can have a key role, in community structure and food web dynamics for the recipient ecosystem^37–40^. Resource subsidies can strongly underpin the observed patterns in biodiversity and overall community composition of recipient ecosystems^41,42^. For example, cross-ecosystem subsidies of organic matter can provide basal resources that are critical for enhancing ecosystem processes and supporting food webs^43,44^. Such resource subsidies are common across terrestrial and aquatic systems^45–47^, principally fueling primary and secondary productivity in recipient ecosystems. In addition to provisioning basal food resources, these cross-ecosystem subsidies also have cascading effects on community structure and the recipient ecosystem food web^48,49^ as well as providing habitat for numerous species^50,51^. They are ubiquitous, ranging from terrestrial organic matter inputs to lakes^52^ and freshwater streams^53^, to carcass and vegetal inputs to the deep ocean benthos^54,55^, and marine macrophyte subsidies to sandy beaches^56,57^.

Characterized by low in situ primary production, sandy beaches are widely distributed intertidal ecosystems with multitrophic food webs that, in many temperate regions, are subsidized by large inputs of marine macrophytes (wrack) from rocky reefs and seagrass beds^47^. Climate change is projected to reduce the subsidy supply in many areas as macrophyte productivity declines due to warming and increased storm disturbance^58,59^. Similarly, the delivery to and retention of wrack on the shoreline of recipient beaches (i.e., receptibility) is decreasing with habitat loss and reduced ecosystem connectivity due to increased storm-driven erosion and sea level rise^60^. Conversely, some regions are projected to see climate change-driven increases in wrack inputs with largely unknown impacts to previously low-subsidy beaches^61–64^. The implications of such changes for the structure and ecological functioning of subsidized ecosystems are poorly understood, especially as these systems demonstrate high levels of spatial synchrony across multiple trophic levels, implying that perturbations to the resource supply would have cascading effects across ecosystem boundaries and through the coastal food web^65^. 

**Fig. 1:**
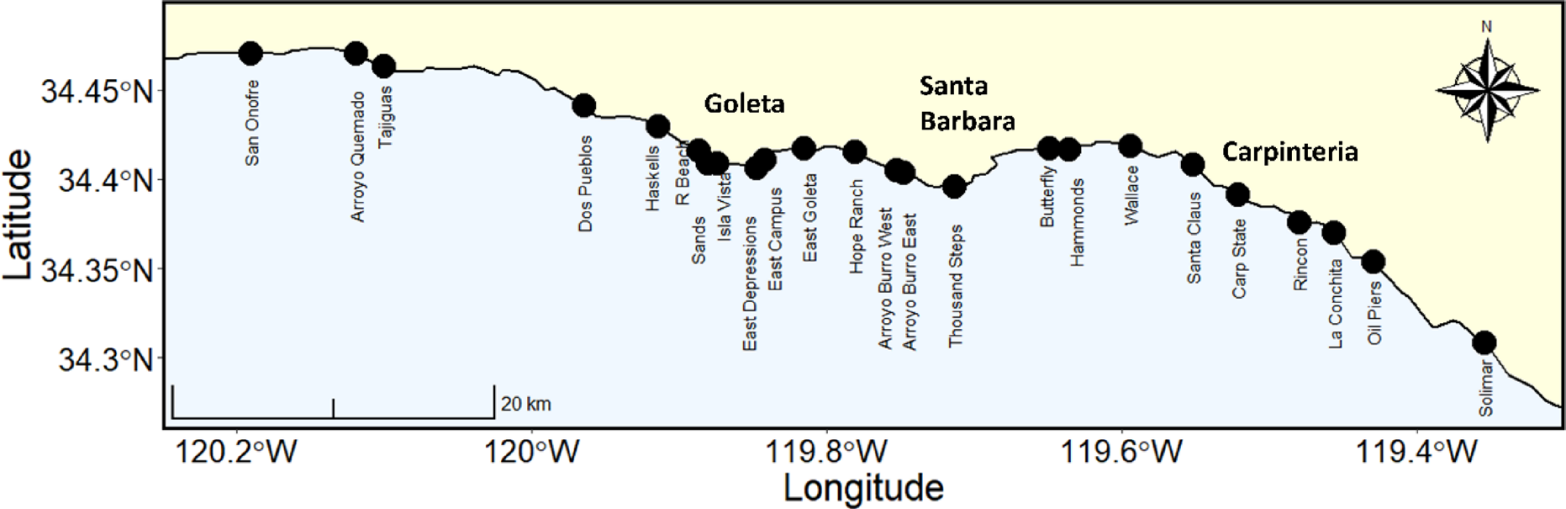
The 24 sandy beach sites sampled in Santa Barbara and Ventura Counties, California, USA.

To evaluate the role of an allochthonous basal resource in structuring food webs and driving ecosystem multifunctionality, we took advantage of a large natural range in the magnitude of wrack inputs to sandy beaches. We utilize a space-for-time approach to develop insights on potential variation in these relationships, via spatial comparisons along environmental gradients, that may occur over time as subsidy dynamics shift^66^. Wrack subsidies, macroinvertebrate and shorebird communities, and multiple ecosystem functions were surveyed at 24 sandy beach sites across ~100 km of Santa Barbara Channel coastline (Fig. 1). We hypothesized that wrack subsidies would strongly drive (1) community structure metrics (diversity, abundance, and biomass) of multiple trophic levels in the food web of the recipient ecosystem, and (2) ecosystem functioning including multifunctionality, based on a diverse suite of important sandy beach ecosystem functions.

## Results

### Subsidy inputs

We measured the cover and composition of wrack on three replicate transects at each study site. Across the 24 sandy beaches, marine macrophyte wrack was compositionally dominated by kelps (29%; Laminariales) and surfgrass (67%; *Phyllospadix spp.*), with the remaining wrack (4%) comprised of miscellaneous macroalgal species. Non-marine wrack was a negligible component of total wrack cover. Average wrack abundance, measured as percent cover, varied widely across the 24 study beaches, ranging from 0.9% to 11.6%.

### Macroinvertebrate community

On the same replicate transects at each study beach, we sampled the wrack-associated macroinvertebrate community. Species richness of macroinvertebrates varied over three-fold among sites with mean values ranging from 4 to 15 species with predatory macroinvertebrates averaging 23.2% of total macroinvertebrate richness (range from 0 to 47.4%) compared to detritivores. Mean values of abundance and biomass of the invertebrate community also varied greatly among sites, ranging from 1,057 to 53,720 individuals m^− 1^ beach and 6.2 to 1389.9 g m^− 1^ beach (blotted wet weight), respectively. Marine wrack abundance was a significant predictor of total macroinvertebrate species richness (r^2^ = 0.58, *p* < 0.0001) and log-transformed abundance (r^2^ 0.19, *p* = 0.02) (Supplemental Fig. 2).

### Ecosystem multifunctionality

For ecosystem functioning, we found high variation in all five standardized individual functions across the 24 study beaches (Supplemental Fig. 1). Nutrient concentrations (total dissolved inorganic nitrogen or DIN) in beach pore water varied across three orders of magnitude, with mean values ranging from 4.8 µM to 2,330.0 µM DIN. The flux of CO_2_ from intertidal sediment at the high tide strand line varied over an order of magnitude among beaches with mean values ranging from 0.09 to 0.35 g CO_2_ m^− 2^ h^− 1^. The secondary production of talitrid amphipods, the dominant macroinvertebrate wrack consumer, and a primary prey resource for higher trophic level consumers, varied over two orders of magnitude, with mean values ranging from 0.07 to 5.84 g m^− 1^ beach day^− 1^ (ash-free dry weight). Flying insect abundance, another primary food source for higher trophic levels, as mean catch per unit effort (number of individuals trapped in 15 min) ranged from less than 1 to 594 individuals. The estimated daily energy requirements of plovers, important consumers of wrack-associated macroinvertebrates, using the study beaches ranged from 0 to 51,278 kJ day^− 1^.

Individual functions were all significantly positively related to wrack abundance, except log-transformed pore water nutrient concentrations (dissolved inorganic nitrogen, r^2^ = -0.01, *p* = 0.42) (Supplemental Fig. 1). Plover daily energy requirements (cubed root-transformed) had the strongest relationship with wrack cover (r^2^ = 0.42, *p* = 0.0004), followed by log-transformed CO_2_ flux (r^2^ = 0.29, *p* = 0.004), log-transformed flying insect catch per unit effort (r^2^ = 0.23, *p* = 0.01, RSE = 0.21), and talitrid secondary production (r^2^ = 0.14, *p* = 0.04) (Supplemental Fig. 1). Ecosystem multifunctionality, based on the five standardized ecosystem functions we measured, ranged from 0.1 to 0.77 (on a scale of 0–1) among beaches.

### Ecosystem response to subsidies

**Fig. 2:**
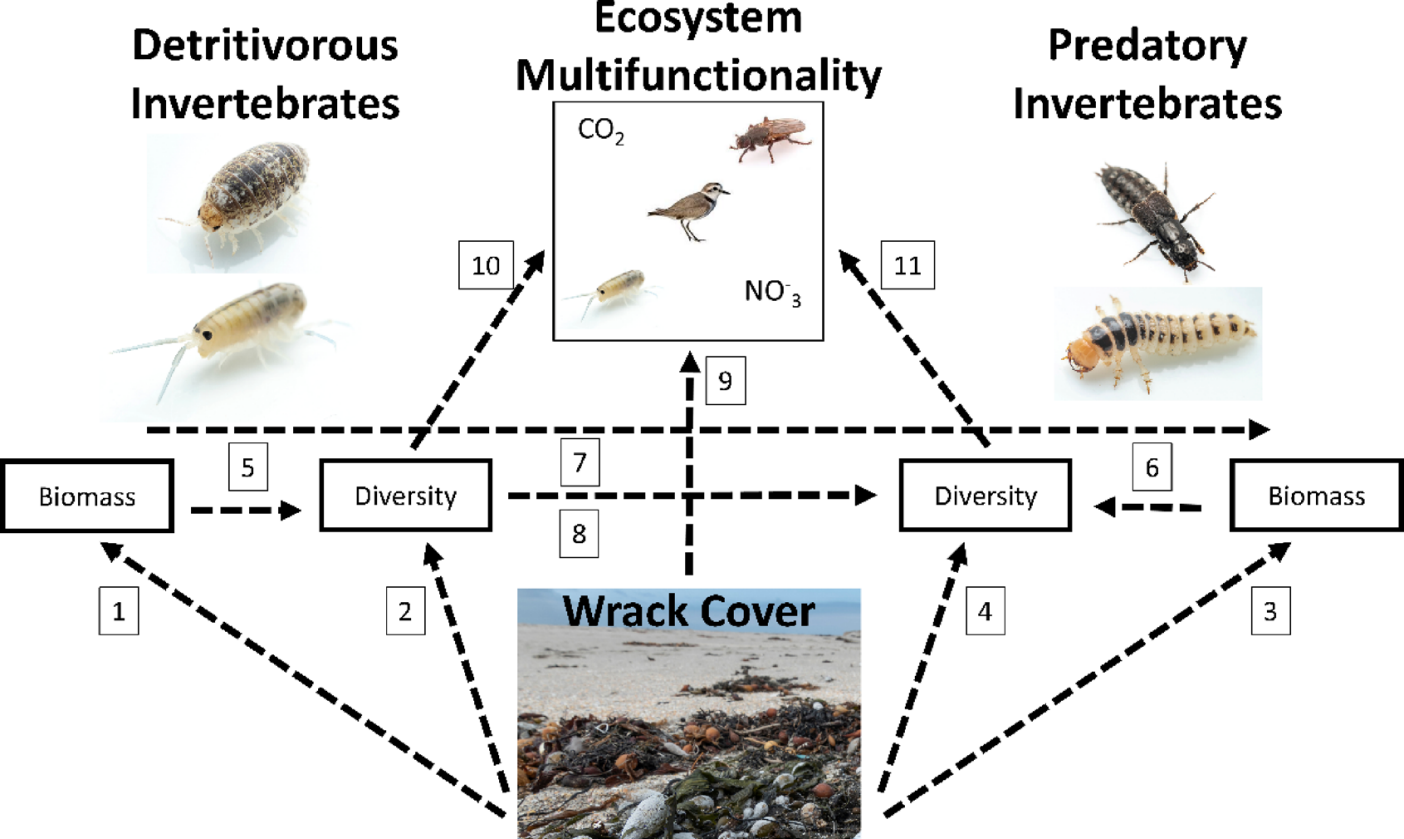
Diagram of 11 hypothesized unidirectional pathways (dashed lines) for ecosystem multifunctionality, macroinvertebrates, and wrack abundance (cover). We hypothesized that wrack subsidies would have direct positive impacts on (1) detritivore biomass, (2) detritivore diversity, (3) predator biomass and (4) predator diversity through the provisioning of food and habitat. We also predicted that (5) detritivore biomass would positively impact detritivore diversity and (6) predator biomass would be associated with greater predator diversity, that (7) detritivore biomass would positively impact predator biomass, that (8) increased detritivore diversity would result in greater predator diversity, and that (9) wrack subsidies, (10) detritivore diversity, and 11) predator diversity would all have positive impacts on ecosystem multifunctionality. PiecewiseSEM does not evaluate reciprocal relationships and it is important to note that variables can be both predictors and responses.

**Fig. 3:**
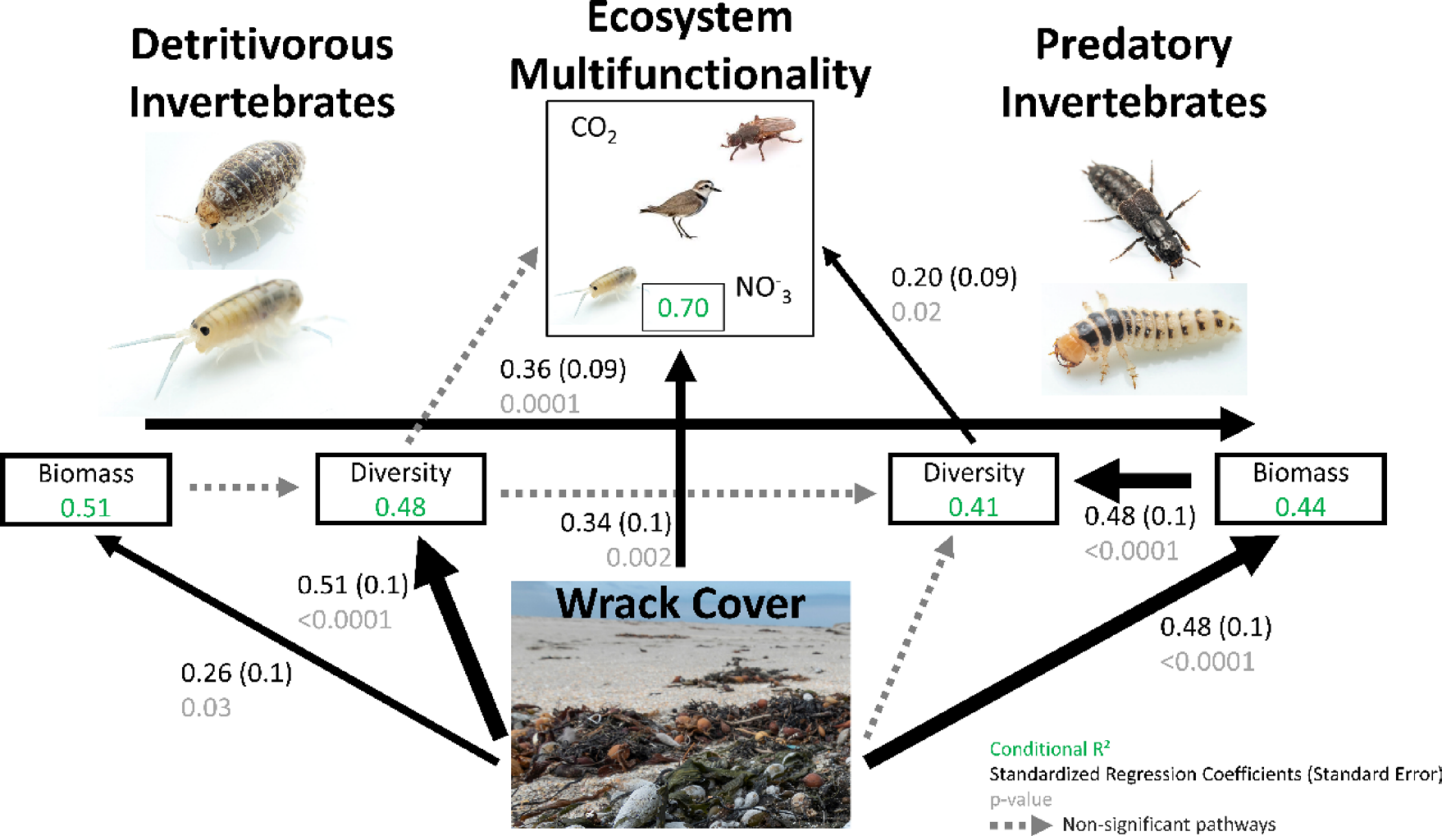
Diagram of PiecewiseSEM model results for ecosystem multifunctionality, macroinvertebrates, and wrack abundance (cover). Significant pathways (p-value (grey)) are displayed with arrows scaled to the size of the standardized regression coefficients (black). Conditional R^2^ values are given for each response variable (green value in boxes). Note that all significant pathways were positive. Non-significant hypothesized pathways are indicated by grey dashed lines. PiecewiseSEM does not evaluate reciprocal relationships and it is important to note that variables can be both predictors and responses. Images courtesy of Michael Ready.

We utilized a piecewise structural equation model (SEM) to evaluate the role of wrack subsidies on the biodiversity and biomass of detritivorous and predatory macroinvertebrates as well as ecosystem multifunctionality. We hypothesized that wrack subsidies would have direct positive impacts through the provisioning of food and habitat (Fig. 2). We also predicted that wrack inputs would impact the relationship between detritivores and predators as well as have positive impacts on ecosystem multifunctionality (Fig. 2). Seven significant pathways were identified among the 11 hypothesized relationships between wrack subsidies and the community structure attributes (Fig. 3). Model results indicated wrack abundance had a strong positive association with detritivore diversity (standardized regression coefficient (SRC) = 0.51, *p* < 0.0001) but not with predator diversity and that wrack abundance was a strong predictor of the biomass of detritivores (SRC = 0.26, *p* = 0.03) and predators (SRC = 0.48, *p* < 0.0001). Detritivore biomass, as hypothesized, positively affected predator biomass (SRC = 0.36, *p* = 0.0001), but detritivore diversity had no effect on predator diversity. Predator diversity was positively associated with predator biomass (SRC = 0.48, *p* < 0.0001), but there was no relationship between detritivore diversity and biomass. Wrack abundance was a strong predictor of ecosystem multifunctionality (SRC = 0.34, *p* = 0.002), as was predator diversity to a lesser extent (SRC = 0.2, *p* = 0.02), but not detritivore diversity. Wrack resource abundance influenced the intertidal macroinvertebrate food web across two trophic levels and multiple ecosystem functions, including strong positive effects on detritivore diversity, the biomass of both detritivores and predators, and ecosystem multifunctionality.

### Shorebird response to subsidies

**Fig. 4:**
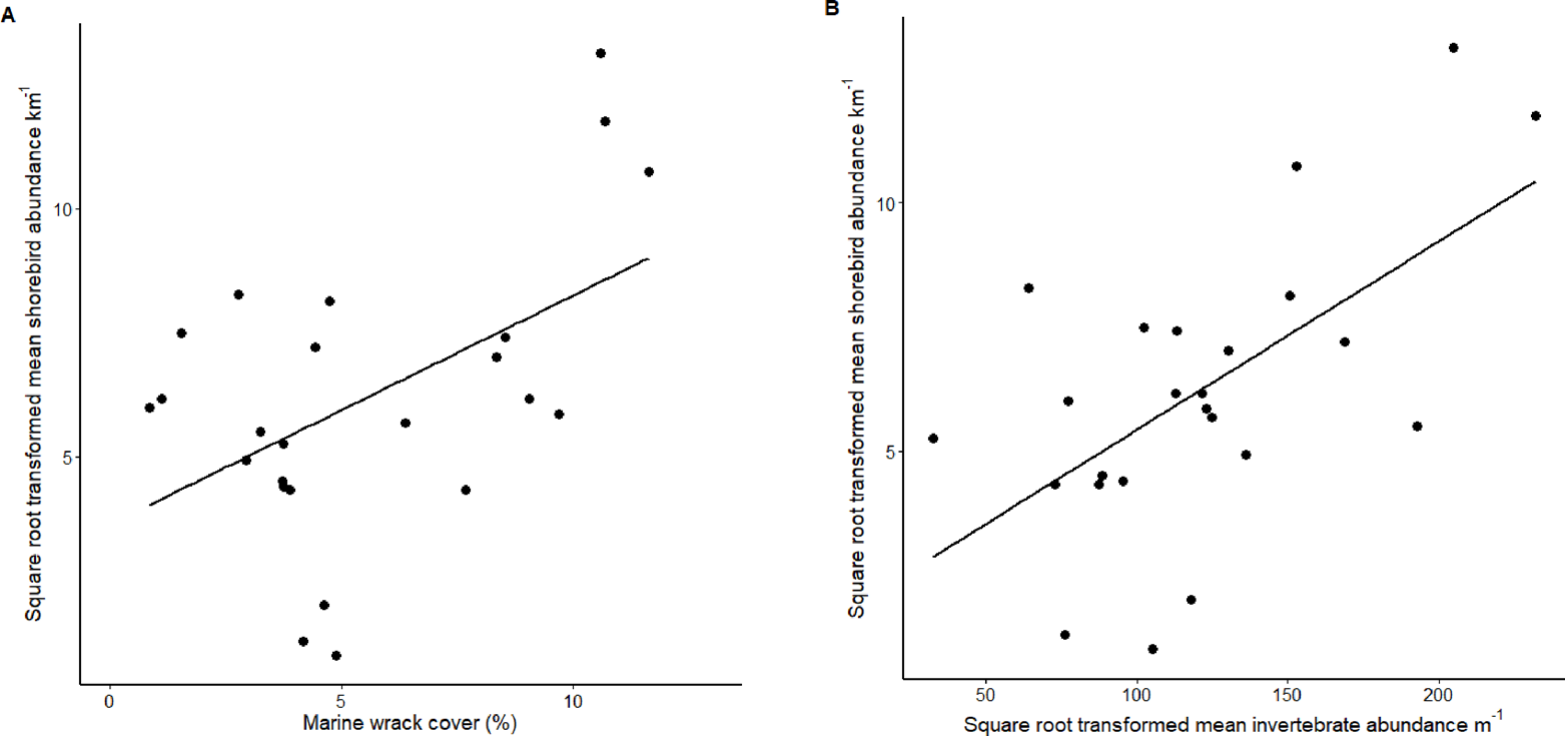
Linear regressions between (**A**) marine wrack percent cover and mean square root-transformed shorebird abundance (individuals km^− 1^ beach) (r^2^ = 0.22, *p* = 0.01) and (**B**) mean square root-transformed macroinvertebrate abundance (individuals m^− 1^ beach) and mean square root-transformed shorebird abundance (r^2^ = 0.33, *p* = 0.002) at our 24 study beaches.

Shorebirds, the most abundant top predators of wrack-associated macroinvertebrates in sandy beach food webs, were surveyed on broader spatial and temporal scales (1 km beach transects for 3 months) because of their greater size and mobility. Mean shorebird richness ranged from 0.7 to 8.2 species km^− 1^ and mean plover richness ranged from 0 to 4 species km^− 1^. Mean shorebird abundance ranged from 1 to 172.4 individuals km^− 1^ and mean plover abundance ranged from 0 to 119.7 individuals km^− 1^. Shorebirds (square root-transformed abundances) were significantly more abundant on beaches with higher wrack abundance (Fig. 4A, r^2^ = 0.22, *p* = 0.01) and more abundant macroinvertebrate (square root-transformed) prey (Fig. 4B, r^2^ = 0.33, *p* = 0.002). The species richness of shorebirds (log-transformed) was also positively correlated with wrack abundance (r^2^ = 0.30, *p* = 0.003), macroinvertebrate species richness (r^2^ = 0.44, *p* < 0.001), and macroinvertebrate abundance (square root-transformed) (r^2^ = 0.30, *p* < 0.003).

## Discussion

Marine foundation species maintain their foundational role as detrital inputs in subsidized ecosystems with little to no in situ primary production. Detrital subsidies are known to impact ecosystem structure and function in recipient ecosystems^67,68^. However, we demonstrate here that these impacts support multiple trophic levels within the recipient ecosystem food web as well as enhance ecosystem multifunctionality. The supply of allochthonous basal resources of marine macrophyte wrack from nearshore rocky reefs and kelp forests was a strong predictor of the structure and function of sandy beach ecosystems. Our results showed that the magnitude of this cross-ecosystem connectivity, quantified here as the abundance of wrack subsidies, directly influenced the structure of multiple trophic levels of the intertidal beach food web, from invertebrate detritivores to predatory shorebirds. The diversity, abundance, and biomass of these key trophic groups were all enhanced by the amount of wrack subsidies delivered to the beach ecosystem. Importantly, wrack subsidies were also strong predictors of ecosystem multifunctionality, an integrative measure of multiple ecosystem functions. The high responsiveness of ecosystem multifunctionality to wrack subsidies highlights the profound influence of these resource inputs on sandy beach ecosystems. Ecosystem productivity as a driver of diversity is a long-standing paradigm^33^ and the similar importance of subsidies indicate that resource supply drives the relationships between productivity and biodiversity or ecosystem functioning^35,36^.

Foundation species, including canopy-forming kelps on rocky reefs^69,70^, seagrasses^71^, salt marsh grasses^72^ and dominant forest trees^73^, have a vital role in ecosystem functioning and structuring communities. Here, we have shown that allochthonous primary production by the marine foundation species, giant kelp (*Macrocystis pyrifera*) and surfgrass (*Phyllospadix spp.*), exerted strong positive effects on macroinvertebrate community structure and ecosystem multifunctionality. Even as detritus, these species continues to function as a foundational species in subsidized recipient ecosystems^74,75^. The structure (habitat) and basal resources supplied by wrack subsidies influence the bottom-up dynamics of sandy beach ecosystems across the globe^47^ with predators at the top of the beach food web relying on prey supported by inputs of marine subsidies^56,76–78^. The cross-ecosystem importance of marine macrophytes as foundation species has implications for understanding the role of ecological connectivity across ecosystems and illustrates the potential influence of donor ecosystems on the potential outcomes of conservation and management efforts to be substantially more far-reaching than is usually considered^78^.

The need to understand and quantify ecosystem functioning of natural communities in an integrative context is growing as climate change and anthropogenic development alter ecosystems across the globe^17,79^. However, most studies have focused on single measures of ecosystem functioning, often primary production^80^, which while informative, constrains what can be inferred about broader ecological processes in ecosystems. Multifunctionality metrics can also have limitations, however, and can elide how the response of individual functions may differ from the aggregate^81^. Nevertheless, multifunctionality provides a robust estimate of an ecosystem’s functional integrity, as well as a system-scale synoptic measure of function that can be used to span broader spatial and temporal scales of comparison^82^ and trace the impacts of climate change and anthropogenic alterations^28,83,84^. Multifunctionality metrics can also help account for uncertainties associated with the measurement or estimation of individual functions. We show how a multifunctionality metric can contribute to an integrative and holistic overview of the relationship between resource supply, community structure, and ecosystem function.

In most ecosystems, the in situ primary production that fuels food webs is perhaps the most important measure of ecosystem functioning^85^. In contrast, subsidized ecosystems rely on strong connectivity with adjacent ecosystems that supply allochthonous basal resources to food webs^86,87^. The vital cross-ecosystem connectivity of sandy beaches with coastal reefs and kelp forests via drift macrophytes can be affected by wave climate, currents, and beach morphology as well as the availability and extent of the nearshore resource supply^65,88,89^. Factors such as hydrologic connectivity between beaches and reefs, metapopulation dynamics of invertebrates across beaches, and the ecogeomorphic condition of the beach must also be considered as potential drivers of the relationships observed in this study. On southern California beaches, a main component of wrack subsidies to beaches is giant kelp (*Macrocystis pyrifera*)^57^. The productivity of giant kelp fluctuates greatly on seasonal and annual timescales due to variability in storm disturbance, recruitment, and growth rates^90^ as well as on longer time scales associated with climate cycles including the North Pacific Gyre Oscillation and ENSO^91^. Much of this productivity is exported out of the kelp forest^92^, drifting and sinking offshore or washing up on the beach as wrack^93^.

As an intrinsic driver of subsidy dynamics in recipient ecosystems, including sandy beaches^89^ and other subsidized systems^53^, variation in the condition of donor ecosystems is an important but often poorly quantified element of connectivity. During a time of rapid environmental change, the dynamics of cross-ecosystem connectivity and its role in trophic and functioning support may be greatly affected, increasing the vulnerability of both donor and recipient ecosystems. Foundation species like giant kelp, which can be the dominant form of net primary production in the nearshore temperate ocean, profoundly influence community structure and ecosystem function on reefs^69^. At the same time, we show here through comparisons of in situ measurements that large subsidies of exported NPP from giant kelp forests also strongly influence recipient sandy beaches, a finding in agreement with experimental studies^94–96^. As impacts to donor ecosystems can cascade to recipient ecosystems, predicted declines of giant kelp carry significant implications not only for subtidal reefs, but also for kelp subsidy-dependent intertidal systems like beaches^65,97^. Combined with loss of beach habitat from sea level rise, erosion and coastal development, the loss of kelp subsidies could result in fundamental ecosystem changes including wildlife declines and species extinctions. Escalating pressures are already driving shifts in species distributions, declines in biodiversity, and reductions to ecosystem functioning in coastal ecosystems^98,99^. As the impacts of climate change continue to imperil coastal ecosystems, increasing our understanding of the role of ecosystem connectivity on ecological structure and functioning will provide needed insights and tools for the conservation of these vulnerable systems and their irreplaceable ecosystem functions.

## Methods

### Beach sampling

The Santa Barbara Channel is a highly productive coastal region in the Southern California Bight characterized by nearshore giant kelp (*Macrocystis pyrifera*) forests growing on subtidal rocky reefs, and a coastline dominated by sandy beaches. We quantitatively surveyed 24 sandy beaches across ~ 100 km of coastline in Santa Barbara and Ventura counties, California, USA (Fig. 1). We selected sites meeting two criteria: (1) a lack of direct human manipulation (e.g., beach grooming and sand nourishment) and (2) an upper beach zone with at least 2 m of dry upper beach above the 24-hour high tide line during our survey period. All beaches were surveyed within two hours of low tide (≤ 0.76 m MLLW) during October and early November 2017, prior to the seasonal onset of larger swells and storms. At each site, measurements and samples were collected on three shore-normal transects that extended from the upper beach limit (cliff base or dune toe) to the top of the swash zone. Transects were randomly placed at least 10 m or more apart using a random number generator (therefore not targeting or avoiding wrack) within a 100 m section of beach. Wrack abundance was measured on each transect using a line-intercept method to estimate wrack cover for a 1.0 m wide band of intertidal habitat^56^. Total percent cover of wrack was calculated by taking the total length of marine wrack deposits measured on each cross shore transect and dividing that value by the beach width, defined as the distance from the upper beach limit to the upper swash zone on each transect.

The diversity, abundance, and biomass of the upper beach macroinvertebrate community was surveyed using a series of 10 cm diameter, 20 cm deep cores along each transect. Transects were split into two zones – the dry upper beach zone and the talitrid amphipod zone. Talitrid amphipods are abundant beach detritivores and occupy the lowest intertidal zone of the wrack-associated upper beach macroinvertebrates. The upper zone extended from the upper beach limit to the top of the talitrid amphipod zone, defined by the upper and lower limit of talitrid burrows on each transect. Within each zone, ten evenly spaced cores were taken and combined into a fine mesh (1.5 mm) bag. Each combined sample was rinsed free of sand in the surf, emptied into labeled one gallon Ziplock bags and frozen. In the laboratory, the samples were defrosted and rinsed into sorting trays with DI water and sorted to separate invertebrates from wrack and sediment. Invertebrates were identified to species level, counted, and weighed to the nearest mg (blotted wet weight).

We measured five ecosystem functions that we predicted would respond to wrack subsidies: pore water nutrient concentration, sediment CO_2_ flux, talitrid amphipod secondary productivity, flying insect abundance, and shorebird (plover) daily energy requirements. Sediment CO_2_ flux and dissolved nutrient concentrations reflect wrack utilization and processing by microbes and invertebrates^57,100^. Biotic functions including secondary production of talitrids, flying insect abundance, and daily energy needs of plovers reflect resource use and availability for higher trophic levels and food web responses^56,101–103^.

To assess nutrient cycling and retention, samples of intertidal pore water were collected for nutrient analyses by digging pits at the high tide line on each transect and allowing pore water to fill the pits before using a 60 ml syringe to sample surface pore water. All pits were located at the same tidal height (the high tide line or highest extent of total water level on the beach in the preceding 24-hour period), but to be consistent across transects and sites the pits were not excavated under wrack deposits. Samples were filtered through glass fiber filters into two 20 ml glass scintillation vials and then frozen. Nutrient analyses of defrosted samples for ammonia and nitrate + nitrite concentrations were conducted at the Marine Science Institute Analytical Laboratory at the University of California, Santa Barbara using simultaneous flow injection analysis on a Lachat QuickChem 8500 Series 2 Analyzer (Hach Company, precision ± 5%).

Intertidal community respiration, as the flux of CO_2_ from the damp sand at the 24-hour high tide line, was measured using a PP Systems EGM-5 coupled to an SRC-2 soil respiration chamber (accurate to < 1% over calibrated CO_2_ range). The chamber was placed on a collar inserted 2 cm into the sand surface at the 24-hour high tide line immediately adjacent to the transect on bare sand rather than directly over surface wrack deposits to ensure consistent measurements across transects and sites and to avoid inflated flux values relative to the broader beach landscape. Flux measurements were made over three minutes and reported as the linear flux rate (g CO_2_ m^− 2^ h^− 1^). This measurement of community-level net respiration includes microbial decomposition and invertebrate respiration. Any primary production offset was assumed to be negligible because measurements were not made over wrack or vegetation and sampling sites were located above the zone where interstitial or surf zone diatoms would be present^104,105^.

The sex of all talitrid amphipods (except juveniles < 5 mm) was recorded and body length was measured for use in estimation of secondary productivity using a length to ash free dry weight (AFDW) relationship and the equation of Edgar^106^. As talitrids are the dominant detritivore on the study beaches (median biomass of invertebrate community = 90%), their secondary productivity represents the majority of production available to higher-level consumers.

Flying insects (Diptera and Coleoptera) are also an important component of the wrack-associated community and food web and are consumed by predatory invertebrates and birds. Since these intertidal insects are not effectively sampled by sediment cores, we measured their abundance using sticky traps. On each transect, two rolls of ribbon fly paper (Revenge, Bonide Products, NY, USA) were unrolled and pinned at each end into the sand with stake flags on top of two different patches of fresh kelp wrack located near the 24-hour high tide line. The sticky traps were deployed undisturbed for 15 min, then collected into labeled one-gallon zip-lock bags and frozen. In the laboratory all flying insects (i.e. *Fucellia* spp., *Coelopa* spp., *Cafius* spp., Staphylinidae spp.) were identified and counted. Abundances from the two traps deployed on each transect were summed and mean abundances calculated as catch per deployment among the three transects.

Shorebirds generally represent the most abundant top predators of wrack-associated macroinvertebrates in intertidal beach food webs and were surveyed at a larger spatial scale (1 km) than invertebrates. Shorebird surveys were conducted at each study site during the initial beach survey and on two additional dates at equivalent low tides between October 2017 and February 2018. Shorebird surveys were conducted during the overwintering period when resident and migratory shorebirds on present on beaches in the region (September – March), we avoided migration periods during the Spring and Summer (April – August)^101,107^. Shorebirds were identified to species and counted on a standard 1 km alongshore transect of beach centered on the location where wrack and invertebrate surveys were conducted. The 1 km transects were contained within the study beach and did not cross onto different beaches. Shorebird abundance was expressed as individuals km^− 1^. Plovers, a common group of shorebirds in the study region, are visual predators that forage on talitrids and other wrack-associated invertebrate prey^56,108^. Because of their strong associated with wrack and wrack-associated invertebrates, we used plovers’ daily energy requirement for each beach as an ecosystem function as it reflects the energy flow from wrack that would be needed to support this group of shorebirds. Plover daily energy requirement (kJ day^− 1^) was calculated based on our field-measured abundances from the replicate shorebird surveys at our 24 study sites in conjunction with species-specific average adult body sizes and metabolic scaling equations for shorebirds^103,109–111^.

### Data analyses

Invertebrate abundance and biomass were scaled based on core spacing to estimate the abundance or biomass of each species m^− 1^ of shoreline^56^. We used Ordinary Least Squares (OLS) linear regression analyses to compare shorebird abundance to wrack cover and macroinvertebrate abundance. Shorebird and macroinvertebrate abundances were square root-transformed to meet the assumptions of OLS linear regression based on Shapiro-Wilk tests for normality, Breusch-Pagan tests for homoscedasticity, and Durbin-Watson tests for autocorrelation of residuals.

We estimated ecosystem multifunctionality by combining multiple ecosystem functions into a unitless, integrative metric using the R package *multifunc*^27^. Ecosystem functions were scaled to values between 0 and 1 based on the maximum observation for each of the five functions we considered, and then the average of these values was used as a site-level estimate of ecosystem multifunctionality. For porewater nutrient concentrations, sediment CO2, and flying insect abundance no assumptions or calculations were made as we used directly measured values. For secondary production we used established secondary productivity allometric relationships for talitrid amphipods from the study region. For shorebird daily energy requirement, we assumed this would be best reflected by the plover community as they are visual foragers that are the predominant predator of talitrids and other wrack-associated invertebrate prey. We compared the relationship between each of the five independent functions and wrack cover using OLS linear regression. Pore water nutrient concentration, sediment CO_2_ flux, talitrid amphipod secondary productivity, and flying insect abundance were log-transformed and shorebird (plover) daily energy requirements were cube root-transformed to meet the assumptions of OLS linear regression based on Shapiro-Wilk tests for normality, Breusch-Pagan tests for homoscedasticity, and Durbin-Watson tests for autocorrelation of residuals.

All sampled macroinvertebrates were classified as one of two functional groups – detritivores or predators. The detritivores were dominated by talitrid amphipods (*Megalorchestia* spp.), but also included isopods (*Alloniscus perconvexus* and *Tylos punctatus*) and beetles (e.g., *Phaleria rotundata* and *Cercyon fimbriatus*), which are also common and widely distributed wrack consumers. Common predators included members of several beetle families (*Caribidae*,* Histeridae*,* Staphylinidae*) and spiders (*Salticidae*). Abundance and biomass were summed by functional group for each transect. Diversity of order 1 for each functional group was calculated for each transect:

$$\:1D=\text{e}\text{x}\text{p}\left(H\right)$$

where H is the Shannon entropy index^112^. Diversity of order 1 is a frequency-weighted value derived from the abundance of each species and does not favor rare or common species^112^.

The role of marine wrack subsidies in structuring the macroinvertebrate food web and ecosystem multifunctionality was evaluated using piecewise structural equation modeling (PiecewiseSEM)^113^. We used marine wrack percent cover along with diversity of order 1 and biomass of both functional groups and the ecosystem multifunctionality metric with hypothesized unidirectional pathways in generalized linear mixed models^114^ with site as a random factor. Predictor variable data were standardized to a mean of 0 and standard deviation of 1^70^. PiecewiseSEM does not evaluate reciprocal relationships and it is important to note that variables can be both predictors and responses^113^. We evaluated the following hypothesized potential pathways between wrack subsidies, two trophic guilds of macroinvertebrates, and ecosystem multifunctionality (Fig. 2): Wrack subsidies would have direct positive impacts on (1) detritivore biomass and (2) detritivore diversity through the provisioning of food and habitat, and wrack subsidies would have direct and indirect positive impacts on (3) predator biomass and (4) predator diversity through the provisioning of habitat and increased prey availability, respectively. We also predicted that (5) detritivore biomass would positively impact detritivore diversity and (6) predator biomass would be associated with greater predator diversity. We predicted that (7) detritivore biomass would positively impact predator biomass as a direct response to increased prey availability and that (8) increased detritivore diversity would result in greater predator diversity as more potential prey types may support more predator species. Lastly, we hypothesized that (9) wrack subsidies, (10) detritivore diversity, and 11) predator diversity would all have positive impacts on ecosystem multifunctionality.

## Supplementary Information

Below is the link to the electronic supplementary material.

Supplementary Material 1

## Data Availability

The data are available through the Environmental Data Initiative (10.6073/pasta/9edd88a04edd7de3a7abe4fe21225f68).
