# Supplementary material for: Food web structure and ecosystem multifunctionality in a subsidized coastal ecosystem
Source: Sci Rep. 2025 Nov 7;15:39086. doi: 10.1038/s41598-025-25395-5 (PMC12594867; doi:10.1038/s41598-025-25395-5)
Supplement: Supplementary file 1 — Supplementary Material 1 [file 41598_2025_25395_MOESM1_ESM.docx]

**Supplemental Material**

**Title**

Food web structure and ecosystem multifunctionality in a subsidized coastal ecosystem

**Format**

Article

**Authors**

Kyle A. Emery*^1,2^, Jenifer E. Dugan^1^, David M. Hubbard^1^, J. Carter Ohlmann^3^, Jessica R. Madden^1^, Robert J. Miller^1^

^1^ Marine Science Institute, University of California, Santa Barbara, Santa Barbara, CA, 93106, USA

^2^ Department of Geography, University of California, Los Angeles, Los Angeles, CA 90095, USA

^3^ Earth Research Institute, University of California, Santa Barbara, Santa Barbara, CA, 93106, USA

*** Corresponding Author:**

Kyle Emery

Marine Science Institute, University of California, Santa Barbara

Santa Barbara, CA 93106 USA

Email: emery@ucsb.edu

Phone: 609-760-5993

Supplemental Figure 1: The relationship between marine wrack abundance and A) total macroinvertebrate species richness (r^2^ = 0.58, p < 0.0001) and B) log-transformed mean macroinvertebrate abundance (r^2^ 0.19, p = 0.02).


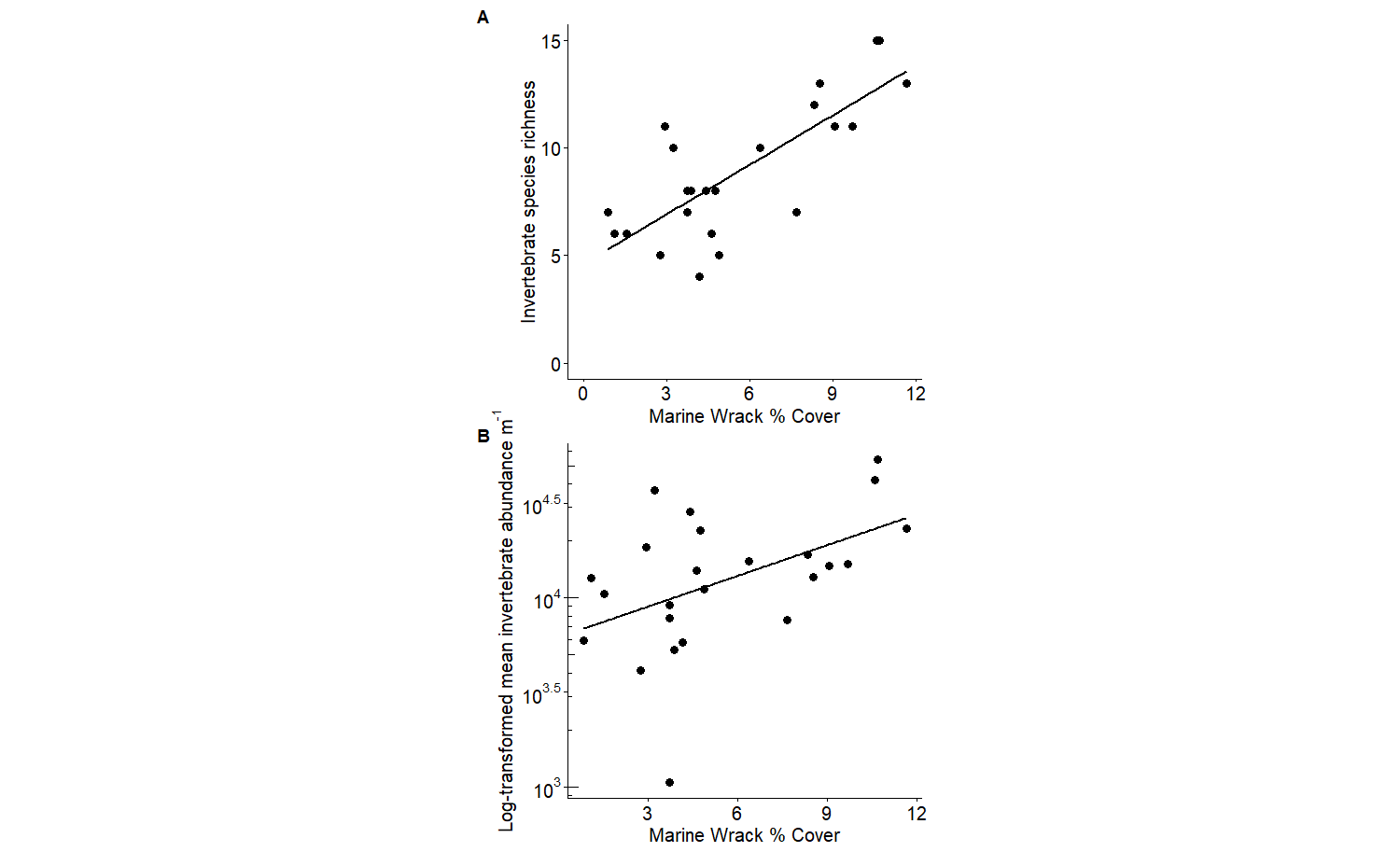


Supplemental Figure 2: Individual ecosystem functions plotted against wrack cover. A) Log-transformed total dissolved inorganic nitrogen in beach pore water (r^2^ = -0.01, p = 0.42). B) Log-transformed CO_2_ flux at the high tide strand line (r^2^ = 0.29, p = 0.004). C) Log-transformed secondary production of talitrid amphipods (r^2^ = 0.14, p = 0.04). D) The cubed root of estimated daily energy requirements of plovers (r^2^ = 0.42, p = 0.0004). E) Log-transformed flying insect abundance as mean catch per unit effort (r^2^ = 0.23, p = 0.01).


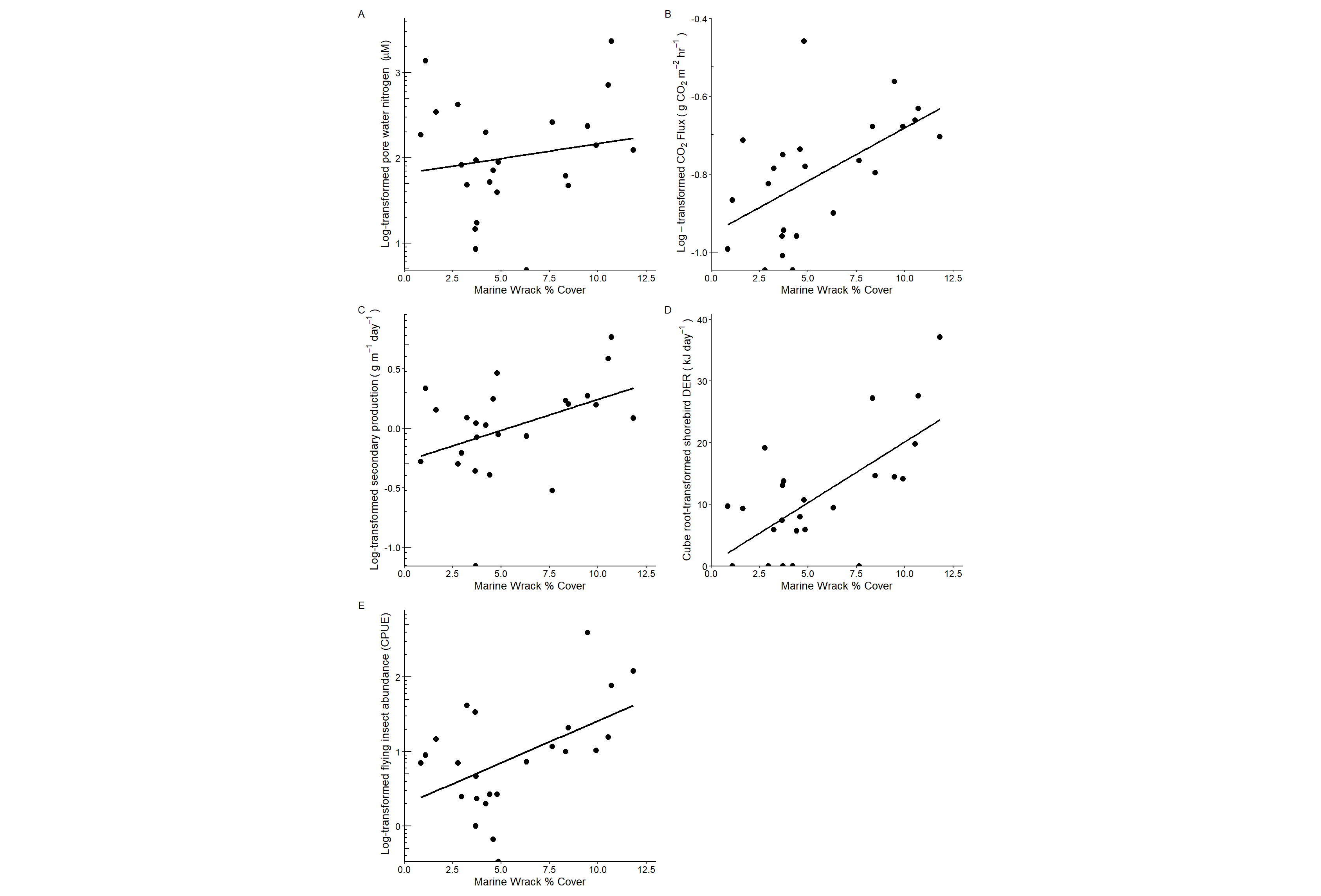


Supplemental Table 1: A species list of all invertebrates collected during beach surveys and the trophic classification (Detritivore or Predator) assigned to each. Unk. refers to unknown. All collected invertebrates belong to the phylum Arthropoda.

| **Class** | **Order** | **Family** | **Genus** | **Species** | **Tropic Group** |
| --- | --- | --- | --- | --- | --- |
| Insecta | Coleoptera | Curculionidae | Emphyastes | fucicola | Detritivore |
| Insecta | Coleoptera | Curculionidae | Thalasselephas | testaceus | Detritivore |
| Insecta | Coleoptera | Curculionidae | Unk. | Unk. A. | Detritivore |
| Insecta | Coleoptera | Curculionidae | Unk. | Unk. B. | Detritivore |
| Insecta | Coleoptera | Hydrophilidae | Cercyon | fimbriatus | Detritivore |
| Insecta | Coleoptera | Tenebrionidae | Coelus | ciliatus | Detritivore |
| Insecta | Coleoptera | Tenebrionidae | Coelus | globosus | Detritivore |
| Insecta | Coleoptera | Tenebrionidae | Epantius | obscurus | Detritivore |
| Insecta | Coleoptera | Tenebrionidae | Phaleria | rotundata | Detritivore |
| Insecta | Diptera | Anthomyiidae | Fucellia | rufitibia | Detritivore |
| Insecta | Diptera | Coelopidae | Coelopa | vanduzeei | Detritivore |
| Insecta | Diptera | Empididae | Unk. | Unk. A. | Detritivore |
| Malacostraca | Amphipoda | Talitridae | Megalorchestia | benedicti | Detritivore |
| Malacostraca | Amphipoda | Talitridae | Megalorchestia | californiana | Detritivore |
| Malacostraca | Amphipoda | Talitridae | Megalorchestia | corniculata | Detritivore |
| Malacostraca | Amphipoda | Talitridae | Megalorchestia | minor | Detritivore |
| Malacostraca | Amphipoda | Talitridae | Traskorchestia | traskiana | Detritivore |
| Malacostraca | Isopoda | alloniscidae | Alloniscus | perconvexus | Detritivore |
| Malacostraca | Isopoda | Tylidae | Tylos | punctatus | Detritivore |
| Arachnida | Pseudoscorpiones | Garypidae | Garypus | californicus | Predator |
| Arachnida | Araneae | Unk. | Unk. | Unk. A. | Predator |
| Insecta | Coleoptera | Anthicidae | Amblyderis | parviceps | Predator |
| Insecta | Coleoptera | Caribidae | Akephorus | marinus | Predator |
| Insecta | Coleoptera | Histeridae | Euspilotus | scissus | Predator |
| Insecta | Coleoptera | Histeridae | Hypocaccus | gaudens | Predator |
| Insecta | Coleoptera | Histeridae | Neopachylopus | sulcifrons | Predator |
| Insecta | Coleoptera | Histeridae | Unk. | Unk. | Predator |
| Insecta | Coleoptera | Staphylinidae | Aleochara | sulcicollis | Predator |
| Insecta | Coleoptera | Staphylinidae | Bledius | fenyesi | Predator |
| Insecta | Coleoptera | Staphylinidae | Cafius | canescens | Predator |
| Insecta | Coleoptera | Staphylinidae | Cafius | lithocharinus | Predator |
| Insecta | Coleoptera | Staphylinidae | Cafius | luteipennis | Predator |
| Insecta | Coleoptera | Staphylinidae | Cafius | seminitens | Predator |
| Insecta | Coleoptera | Staphylinidae | Hadrotes | crassus | Predator |
| Insecta | Coleoptera | Staphylinidae | Omalium | algarum | Predator |
| Insecta | Coleoptera | Staphylinidae | Pontomalota | opaca | Predator |
| Insecta | Coleoptera | Staphylinidae | Tarphiota | geniculata | Predator |
| Insecta | Coleoptera | Staphylinidae | Thinopinus | pictus | Predator |
